# Nationwide analysis of prehospital tranexamic acid for trauma demonstrates systematic bias in adherence to treatment guidelines: a retrospective cohort study

**DOI:** 10.1097/JS9.0000000000000712

**Published:** 2023-09-15

**Authors:** Ateeq Almuwallad, Elaine Cole, Andrea Rossetto, Karim Brohi, Zane Perkins, Ross Davenport

**Affiliations:** a Centre for Trauma Sciences, Blizard Institute, Queen Mary University of London; b Barts Health National Health Service Trust, London, UK; c Emergency Medical Services Department, Faculty of Applied Medical Sciences, Jazan University, Kingdom of Saudi Arabia

**Keywords:** haemorrhage, injury, major trauma, older patient, prehospital TXA, sex

## Abstract

**Background:**

Prehospital (PH) tranexamic acid (TXA) improves survival from trauma haemorrhage. Injury mechanism, physiology, and sex demographics vary with patient age. The authors hypothesised that these factors influence TXA guideline compliance and examined national trends in PH use to identify any systematic biases in bleeding management.

**Materials and methods:**

The UK Trauma Audit and Research Network data for TXA eligible patients admitted to major trauma centres were divided into two cohorts: 2013–2015 (*n*=32 072) and 2017–2019 (*n*=14 974). Patients were stratified by PH, emergency department or no TXA use. Logistic regression models explored interaction between PH variables and TXA administration. Results are presented as odds ratios with a 95% CI.

**Results:**

PH TXA use increased from 8% to 27% over time (*P*<0.001). Only 3% of eligible patients who fell less than 2 m received PH TXA versus 63% with penetrating injuries (*P*<0.001). Older patients eligible for PH TXA were less likely to receive it compared to younger patients [≥65 years old: 590 (13%) vs. <65 years old: 3361 (33%), *P*<0.001]. There was a significant interaction between age and sex with fewer older women receiving PH TXA. In shocked patients, one third of females compared to a fifth of men did not receive TXA (*P*<0.001). There was a decrease in PH TXA use as age increased (*P*<0.001).

**Conclusions:**

Despite a threefold increase in use, treatment guidance for PH TXA is not universally applied. Older people, women, and patients with low energy injury mechanisms appear to be systematically under treated. Training and education for PH providers should address these potential treatment biases.

## Introduction

Highlights

- Prehospital (PH) use of tranexamic acid (TXA) was independently associated with younger patients, penetrating injury, shock, a low Glasgow coma score and the presence of a PH physician on scene.
- Older people, women and patients with low energy injury mechanisms are systemically under treated in PH care with respect to the early administration of TXA.
- Despite a threefold increase in the use of TXA in PH care treatment guidelines are not universally applied to all at risk groups for traumatic haemorrhage.

Haemorrhage after major injury accounts for 40% of overall trauma mortality^1–3^. Patients with major bleeding die early with almost three-quarters of bleeding trauma patients dying within the first 6 h of injury, either prehospital (PH) or upon arrival at hospital^4^. Acute Traumatic Coagulopathy occurs within minutes after injury, exacerbates bleeding and is associated with worse clinical outcomes after major trauma^5,6^. Hyperfibrinolysis is a key component of post-traumatic coagulopathy^5^ and can be effectively treated with pharmacological interventions such as the antifibrinolytic drug, tranexamic Acid (TXA)^7^. Currently, TXA is the only medication available for the treatment of haemorrhage, that in clinical trials has been found to reduce mortality when administered in PH care^8,9^ or in the early phase of in-hospital trauma resuscitation^10^. It is therefore considered a vital component of trauma care for bleeding patients in both civilian and military settings, and as such is included on the WHO list of essential medications^11^. However, recent reports have suggested that even in mature trauma systems it is not universally provided to all patients at risk of haemorrhage^12,13^.

A meta-analysis of the CRASH-2 & WOMAN trials of TXA for trauma and postpartum haemorrhage, respectively, reported a 10% reduction in survival benefit for every 15 min delay in treatment^14^. Similarly, in the secondary analysis of the Study of Tranexamic Acid During Air Medical and Ground Prehospital Transport (STAAMP) trial, only when TXA was administered within 1 h of injury, or to shocked patients, was there a reduction in 30-day mortality^9^. Following publication of the CRASH-2 trial in 2010^10^, the administration of PH TXA was approved in the UK by the Joint Royal Colleges Ambulance Liaison Committee (JRCALC) in 2012^15^ and similarly, the American College of Surgeon now endorse the PH administration of TXA^16^. However, many trauma patients fail to receive TXA early after injury, with an apparent sex disparity in use evident in both PH care and the emergency department (ED) from a recent study of the national trauma system of England and Wales^12^. Mechanisms of injury and patient demographics change with increasing age^17^ and decisions whether or not to administer TXA at the scene may be consciously or unconsciously influenced by certain patient factors^18^.

The PH phase of care is an opportunity to deliver time critical interventions for haemorrhage, but it is unknown which clinical factors (patient and injury) at the scene influence clinician decision-making to administer TXA. Hence, it is important to characterize the missed opportunities for a clinically effective pharmacological intervention, in order to target education and training programme for PH care providers. The overall objective of this study was to evaluate the real-world use of PH TXA across a national trauma system in patients who survive to reach the hospital. Firstly, we wished to determine whether the introduction of national guidance led to an improvement in the delivery of PH TXA, and assess any guideline implementation effect. Second, in a contemporary cohort, we aimed to quantify the delays to administration and characterise the injury, demographic, and physiologic phenotypes of patients who received (and did not receive) PH TXA in the context of national treatment guidelines. Finally, we specifically examined the patient subgroups most likely to derive benefit from early TXA treatment (e.g. evidence of active haemorrhage and requiring blood transfusion) to determine any clinical characteristics or patient cohorts which have lower rates of PH TXA administration. We hypothesised that it is possible to identify patients who fail to receive PH TXA using demographic or clinical parameters available at the scene, which may offer a potential opportunity to target improvements in the PH care of bleeding trauma patients.

## Materials and methods

We retrospectively analysed data from the UK Trauma Audit and Research Network (TARN) submitted by all 27 Major Trauma Centres in England and Wales over 7 years, between January 2013 and December 2019.

### Study population

TARN submissions are mandatory for all major trauma centres in England and Wales with data quality assured and validated against Hospital Episode Statistic data. It collects data on trauma patients admitted for three or more days to the hospital or require critical care admission. Deaths at the scene are excluded from the registry as are single-system frailty fractures. TXA was introduced for PH administration by paramedics in 2012 after approval by JRCALC. Indications for TXA administration in trauma patients are: suspected or active blood loss, administration of blood component transfusion, and/or presence of any sign of shock [systolic blood pressure (SBP) ≤90 mmHg or heart rate ≥110 b/m]. UK Best Practice Tariff incentivizes clinicians to administer TXA within one hour from injury^19^ with compliance monitored via TARN. Dosing guidance was standardised according to the CRASH-2 trial protocol^10^ with a 1 g intravenous bolus administered followed by a 1 g intravenous infusion (in-hospital). For the purpose of this study, all patients eligible for TXA administration were defined using the following TARN criteria: haemorrhage control operation (Supplemental Table 1, Supplemental Digital Content 1, http://links.lww.com/JS9/A988) and/or blood component transfusion within 24 h of injury. The location of TXA use (PH care or within the ED) was recorded by administering clinicians. Paediatric patients (<16 years), pregnant women, and patients who were transferred to a major trauma centre from another hospital or suffered burns on more than 5% of the total body surface area were excluded. Regarding ethics approval, TARN has Health Research Authority approval (Patient Information Advisory Group, Section 251) for research on the anonymized data it holds from NHS Trusts. The study was reported following the strengthening the reporting of cohort, cross-sectional, and case–control studies in surgery (STROCSS) guidelines^20^ (Supplemental Digital Content 3, http://links.lww.com/JS9/A990).

### Data collection

All data was provided by TARN and included demographics, injury patterns, vital parameters (PH and ED), time intervals (time to TXA and time to arrival), mode of arrival, TXA administration and location. Outcomes included red blood cell (RBC) administration within 24 h, mortality at 24 h and at 28-days. Patients were divided into two study time periods (2013–15 vs. 2017–19), to examine the early versus late phases of JRCALC PH TXA guidance implementation. In each time period, patients were categorised into three administration groups: PH TXA, ED TXA, and No TXA. Subgroup analysis for patients most likely to benefit from TXA, for example shocked and/or received one or more units of RBCs transfusion was predefined.

### Statistical analysis

Data analysis was performed using IBM SPSS v25 and Graph Pad Prism v8. According to Kolmogorov–Smirnov tests continuous data was nonparametric and therefore compared with Mann–Whitney *U* tests and reported as median and interquartile range. Categorical variables were compared using *χ* ^2^ tests and reported as frequencies and percentages. *χ* ^2^ for trend tests were reported when comparing the negative trend of PH TXA among age and shock categories. Shock was defined as a PH SBP less than or equal to 90 mmHg and/or PH heart rate greater than or equal to 110/min prior to any interventions.

To explore the relationship between age, sex, and PH TXA use, the odds for PH TXA administration in male and female subgroups were calculated. The odds ratio (OR) between sexes was derived for ages covering the full range of the cohort (20, 40, 60, 80, and 100 years; Supplemental Table 2, Supplemental Digital Content 1, http://links.lww.com/JS9/A988). A *P*-value <0.05 was considered statistically significant.

A multivariable logistic regression analysis was conducted to examine the association between PH TXA administration and clinically relevant variables available at within PH care: patient age and sex, mechanism of injury, PH SBP, PH Glasgow coma score (GCS) and PH clinician (air ambulance doctor or land ambulance paramedic). Variables with a *P*-value <0.10 in univariate analysis were entered into the final multivariable models. Model fit was assessed using Hosmer–Lemeshow test. Results are presented as OR with a 95% CI.

## Results

Overall, 51 310 TARN patients who were eligible for TXA were included in the study. These were subdivided into two equal time periods to quantify the TXA guideline implementation effect following changes to treatment guidelines: January 2013 to December 2015 (*n*=32 072) and January 2017 to December 2019 (*n*=14 974). There was a threefold increase in PH TXA administration during the study period from 8% (2013–15) to 27% (2017–19), (*P*<0.001). Across the time periods, PH TXA use was more likely to be in younger, male patients (Table 1). In both study periods, less than 5% of patients who sustained falls less than 2 m received PH TXA, whereas administration in penetrating injuries rates rose from 14 to 26% (Table 1). PH times and injury severity were broadly similar between the early and later cohorts (Table 1), although the proportion of shocked patients decreased from 55 to 45% (*P*<0.001). Time from injury to TXA administration reduced from 51 (35–70) min in 2013–15 to 43 (31–61) min in 2017–19, (*P*<0.001).

**Table 1:** Patient and injury characteristics.

	2013–15	2017–19		2013–15	2017–19		2013–15	2017–19	
	PH TXA *n*=2411	PH TXA *n*=3979	*P*	ED TXA *n*=2899	ED TXA *n*=2737	*P*	No TXA *n*=26762	No TXA *n*=8258	*P*
Age^+^	38 (25–54)	39 (26–56)	0.010	40 (26–59)	49 (29–72)	<0.001	59 (42–78)	56 (35–74)	<0.001
Male	1850 (77)	3129 (79)	0.075	2195 (76)	2008 (73)	0.043	13 823 (52)	5421 (66)	<0.001
Female	561 (23)	850 (21)	<0.001	704 (24)	729 (27)	<0.001	12 939 (48)	2837 (44)	<0.001
MOI			<0.001			<0.001		676 (8)	<0.001
Penetrating	347 (14)	1025 (26)		492 (17)	600 (22)		683 (3)	3587 (43)	
Fall <2 m	62 (3)	147 (4)		224 (8)	568 (21)		15 463 (58)	844 (10)	
Fall >2 m	218 (9)	370 (9)		490 (17)	371 (14)		2736 (10)	2376 (29)	
RTC	1651 (69)	2235 (56)		1524 (53)	1018 (37)		6408 (24)	775 (9)	
Others	133 (6)	202 (5)		169 (6)	180 (7)		1472 (6)		
ISS^+^	22 (11–34)	25 (13–38)	<0.001	22 (13–34)	21 (11–30)	0.016	9 (9–10)	10 (9–20)	<0.001
AIS head ≥3	590 (25)	1052 (26)	0.081	728 (25)	755 (28)	0.035	1762 (7)	1586 (19)	<0.001
PH SBP <90 mmHg	449 (22)	849 (25)	0.012	323 (16)	289 (15)	0.223	593 (3)	266 (7)	<0.001
PH HR ≥110 /m	763 (34)	1399 (38)	0.005	620 (28)	529 (25)	0.027	1835 (10)	659 (14)	<0.001
GCS <8	455 (19)	806 (21)	0.137	434 (15)	344 (13)	0.012	503 (2)	390 (5)	<0.001
Mode of arrival			<0.001			<0.001		5755 (70)	<0.001
Ambulance	1417 (59)	2526 (64)		2052 (71)	2072 (76)		18 902 (71)	524 (6)	
HEMS	990 (41)	1086 (36)		416 (14)	376 (14)		965 (4)	1979 (24)	
Other	4 (0)	371 (1)		431 (15)	289 (11)		6895 (26)		
Time from injury to admission (minutes)^+^	95 (74–95)	89 (67–114)	<0.001	81 (59–111)	87 (62–122)	<0.001	101 (75–143)	115 (79–222)	<0.001
Time from injury to TXA (minutes)^+^	51 (35–70)	43 (31–61)	<0.001	125 (85–184)	141 (95–248)	<0.001	–	–	–

Data presented as^+^ median (IQR), otherwise *n* (%).

AIS, abbreviated injury scale; GCS, Glasgow coma scale; HEMS, helicopter emergency medical services; ISS, injury severity score; MOI, mechanism of injury; PH HR, prehospital heart rate; PH SBP, prehospital systolic blood pressure; RTC, road traffic collision; TXA, tranexamic acid.

**Figure 1:**
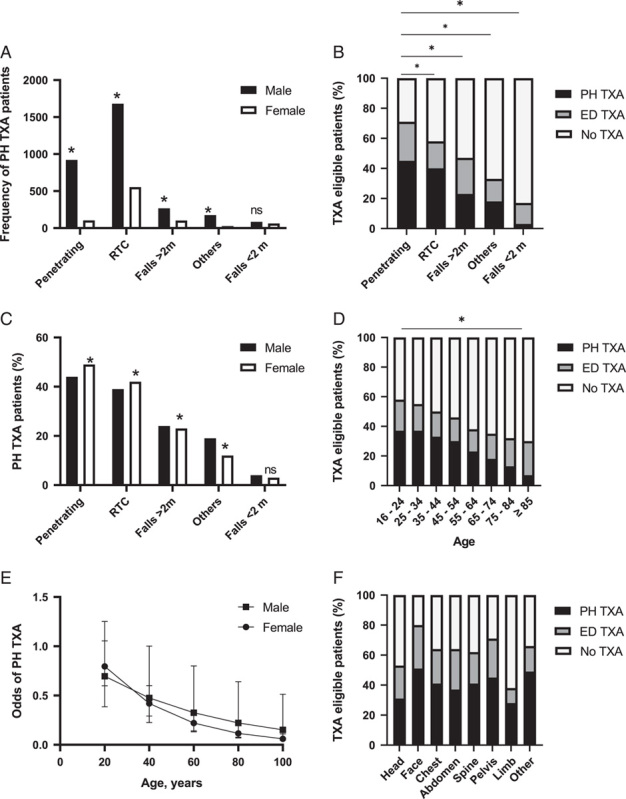
(A) Frequency of PH TXA use across mechanism of injury according to patient sex; *signifies *P*-value <0.001. (B) Percentage patients eligible to receive TXA in mechanism of injury group; *signifies *P*-value <0.001 when comparing PH TXA in penetrating versus other types of mechanism of injury. (C) percentage of PH TXA use across mechanism of injury according to patient sex; *signifies *P*-value <0.001. (D) Percentage patients eligible to receive TXA in age groups; *signifies *P*-value <0.001 when measuring the negative trend of PH TXA with age increased. 1E: Odds of PH TXA for males and females were reported for value of age between 20, 40, 60, 80, and 100 years. 1F: Percentage of PH TXA use across injury groups.

To examine contemporary practice, we focused the investigation on the more recent cohort (2017–2019) to identify individual factors associated with TXA administration. The use of PH TXA varied according to the mechanism of injury, with sex differences observed in penetrating trauma [male: 921 (29%) vs. female: 102 (12%), *P*<0.001] and a similar trend in falls less than 2 m [male: 84 (3%); female: 63 (7%) *P*=0.083] (Fig. 1A). TXA treatment was associated with high energy mechanisms, and almost three-quarters of patients with penetrating injury received TXA, with 63% administered in PH care (Fig. 1B). However, only 3% of patients who fell less than 2 m and were eligible for TXA received it in PH care, and of these only 17% of patients received TXA at any time point (Fig. 1B). When examining the effect of sex on the mechanism of injury, more females received PH TXA after penetrating trauma or road traffic collisions compared to men (Fig. 1C). In contrast, females were less likely to receive it if they had sustained injury by any other mechanism.

Older patients eligible for PH TXA were less likely to receive it compared to younger patients [≥65 year old: 590 (13%) vs. <65 year old: 3361 (33%), *P*<0.001] (Fig. 1D). In both male and female patients, PH TXA treatment was significantly lower with advancing age, but more pronounced in older females (Fig. 1E). In patients aged 45 years or more who were eligible for TXA, between 15 and 40% fewer women received PH TXA compared to men (Fig. 1E), and by 80 years of age, females were half as likely to receive it [OR: 0.524, (95% CI: 0.305–0.899)] (Fig. 1E). Patients with severe (AIS 3+) limb or head injury had the lowest proportion of PH TXA administration, 28 and 31%, respectively (Fig. 1F) and less than 50% of patients with these injuries received TXA in any setting. In multivariable analysis, sex was not associated with PH TXA use [OR 1.168 (95% CI: 0.980–1.393) *P*=0.08] whereas younger age and low PH SBP were strongly predictive of TXA administration [Age OR 0.981 (95% CI: 0.978–0.984), *P*<0.001; SBP: OR 0.992 (95% CI: 0.990–0.994), *P*<0.001] (Table 2).

**Table 2:** Prehospital factors associated with TXA administration.

	Unadjusted OR (95% CI)	*P*	Adjusted OR (95% CI)	*P*
Age	0.982 (0.980–0.984)	0.000	0.981 (0.978–0.984)	0.000
Male sex	1.336 (1.193–1.497)	0.000	1.168 (0.980–1.393)	0.083
Blunt injury	1.789 (1.704–1.885)	0.000	1.601 (1.496–1.728)	0.000
PH SBP	0.991 (0.989–0.993)	0.000	0.992 (0.990–0.994)	0.000
PH GCS	0.946 (0.934–0.957)	0.000	0.955 (0.938–0.973)	0.000
Air ambulance	0.997 (0.997–0.998)	0.000	4.180 (3.357–5.204)	0.000

GCS, Glasgow Coma Scale; OR, odds ratio; PH, prehospital; SBP, systolic blood pressure.

Hosmer–Lemeshow goodness of fit: *X*²: 51.198, *P*<0.001.

**Figure 2:**
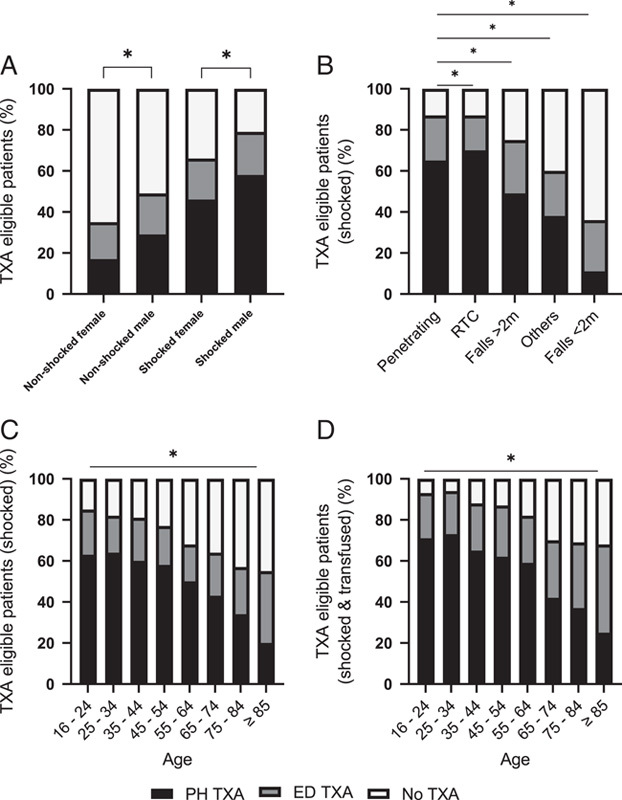
TXA use in eligible patients (percentage values all figure). (A) TXA administration in all eligible patients separated by shock status and sex; *signifies *P*-value <0.001 when comparing PH TXA in shocked (male vs. female) and nonshocked (male vs. female). (B) TXA administration in all shocked eligible patients in mechanism of injury groups; *signifies *P*-value <0.001 when comparing PH TXA in penetrating versus other types of mechanism of injury. (C) TXA administration in all shocked eligible patients in age groups; *signifies *P*-value <0.001 when measuring the negative trend of PH TXA with age increased. (D) TXA administration in all shocked eligible patients having received RBC transfusion in age groups. *signifies *P*-value <0.001 when measuring the negative trend of PH TXA with age increased.

Shocked patients were twice as likely to receive PH TXA than nonshocked patients (54 vs. 24%, *P*<0.001) (Table 3). In the shocked cohort, one third of females compared to a fifth of men did not receive TXA (*P*<0.001, Fig. 2A). Only 11% of patients who were shocked after a fall of less than 2 m received PH TXA, whereas this rose to 65% in penetrating injury with shock (*P*<0.001, Fig. 2B). There was a step-wise decrease in PH TXA use as age increased (*P*<0.001, Fig. 2C) and this trend persisted in older patients with clinical evidence of major haemorrhage and the need for a blood transfusion (*P*<0.001, Fig. 2D).

**Table 3:** Patient and injury characteristics in shock and transfusion cohorts, 2017–2019.

	All patients 14 974	Nonshocked *n*=6816	Shocked *n*=3523	*P*	Shocked + no RBCs *n*=2371	Shocked + RBCs *n*=1150	*P*
Age^+^	70 (103–50)	54 (34–73)	43 (27–61)	<0.001	43 (28–60)	43 (27–63)	0.742
Male	10558 (71)	4630 (68)	2606 (74)	<0.001	1762 (74)	842 (73)	0.487
Female	4416 (29)	2186 (32)	917 (26)	<0.001	609 (26)	308 (27)	<0.001
MOI				<0.001			0.419
Penetrating	2301 (15)	832 (12)	798 (23)		530 (22)	268 (23)	
Fall <2 m	4302 (27)	2324 (34)	598 (17)		414 (18)	184 (16)	
Fall >2 m	1585 (11)	713 (11)	382 (11)		250 (11)	132 (12)	
RTC	5629 (38)	2508 (37)	1512 (43)		1011 (43)	500 (44)	
Others	1157 (8)	439 (6)	233 (7)		166 (7)	66 (6)	
ISS^+^	26 (16–75)	16 (9–25)	22 (10–36)	<0.001	22 (10–35)	24 (13–36)	0.123
AIS Head ≥3	3393 (23)	1632 (24)	1026 (29)	<0.001	694 (29)	331 (29)	0.765
PH SBP <90 mmHg	1404 (9)	0 (0)	1404 (43)	<0.001	882 (40)	522 (50)	<0.001
PH HR ≥110 b/m	2587 (17)	0 (0)	2587 (74)	<0.001	1806 (77)	779 (87)	<0.001
GCS <8	1540 (11)	459 (7)	676 (19)	<0.001	413 (18)	263 (23)	<0.001
Mode of arrival				<0.001			0.058
Ambulance	10343 (69)	5734 (84)	2615 (74)		1786 (75)	827 (72)	
HEMS	2330 (16)	1066 (15)	902 (27)		580 (25)	322 (28)	
Others	2301 (15)	16 (0)	6 (0)		5 (0)	1 (0)	
Time from injury to admission (minutes)^+^	143 (99–1434)	97 (73–128)	87 (64–116)	<0.001	88 (64–117)	85 (65–113)	0.182
Time from injury to TXA (minutes)^+^	124 (61–1406)	72 (42–133)	51 (34–89)	<0.001	50 (33–85)	52 (35–93)	0.065
PH TXA	3979 (27)	1643 (24)	1918 (54)	<0.001	1230 (52)	687 (60)	<0.001

Data presented as^+^median (IQR), otherwise *n* (%).

AIS, abbreviated injury scale; GCS, Glasgow coma scale; HEMS, helicopter emergency medical services; ISS, injury severity score; MOI, mechanism of injury; PH HR, prehospital heart rate; PH SBP, prehospital systolic blood pressure; PH TXA, prehospital Tranexamic Acid; RBCs, red blood cells.

Overall time to TXA dosing reduced after implementation of the treatment guidelines but only 49% of patients eligible for TXA received it within 1 h of injury (Table 1). The time from injury to TXA dosing was faster in shocked patients [51 (34–89) min] compared to the nonshocked [72 (42–133) min, *P*<0.001]. Three-quarters of shocked patients received the drug in PH care (Table 3, Supplemental Figure 1A and 1B, Supplemental Digital Content 2, http://links.lww.com/JS9/A989). The time of TXA dosing in PH care or the ED was fastest for shocked patients with penetrating injuries whereas patients with shock following a fall less than 2 m experienced the longest delays to treatment (Supplemental Figure 1C and 1D, Supplemental Digital Content 2, http://links.lww.com/JS9/A989). Time to PH TXA treatment for shocked patients who had fallen less than 2 m was on average 59 min compared to 45 min for other blunt mechanisms of injury (*P*<0.001) (Supplemental Figure 1C, Supplemental Digital Content 2, http://links.lww.com/JS9/A989). Similarly, TXA administration in the ED was one and a half times longer for low-level falls for patients with shock compared to other blunt injuries (212 min vs. 125 min, *P*<0.001) (Supplemental Figure 1D, Supplemental Digital Content 2, http://links.lww.com/JS9/A989).

## Discussion

In this longitudinal study of all major trauma patients admitted to hospital, within a national trauma system, we examined the clinical characteristics and patient physiology associated with the PH use of TXA following the implementation of trauma haemorrhage treatment guidelines. Overall, PH TXA treatment increased threefold from the period after publication of the CRASH-2 trial and adoption of TXA into UK ambulance service protocols and National Institute for Health and Care Excellence (NICE) Guidelines^10,21–23^. In England and Wales between 2017 and 2019, we found only half of TXA eligible patients (according to TARN criteria) were treated, of which 60% was given in PH and less than half were dosed within 1 h of injury. PH TXA use was independently associated with younger patients, penetrating injury, shock, low GCS and the presence of a HEMS team on scene. While we confirmed similar sex inequities in TXA use, with elderly women far less likely to receive TXA in PH care^12^, we further identified gross treatment disparities in other patient groups. TXA treatment was significantly less common in older patients, those who had sustained low energy falls or patients without clinical evidence of haemorrhagic shock. Real-world practice has identified a large cohort of patients that would benefit from early (PH) TXA administration, whom currently do not receive it despite national guidelines for haemorrhage management^15,21^, and should be the focus of education programmes to improve treatment compliance and patient outcomes.

Identification of bleeding in older patients with injury is confounded by an altered physiological response to blood loss^24^ and polypharmacy^25^. More recently we have shown that the coagulation response to injury of older patients differs to younger patients^26^, and may in part explain the increased incidence of major haemorrhage previously reported in this subgroup^27^. A recent systematic review argues that older major trauma patients are at risk of under triage in PH care and Major Trauma Centres, which may result in less intervention and active management^28^. Our study provides further evidence to support this observation in older patients, and/or those who have fallen less than 2 m, who are far less likely to receive TXA despite clinical evidence of shock. Moreover, we show the PH TXA treatment is not only dependent on age, but that with increasing age women are less likely than men to receive it. This supports the finding of Nutbeam *et al*.^12^, confirming the presence of a sex bias in the treatment of patients in PH care despite clinical evidence of shock, with the use of TXA unequally distributed amongst patients based on sex, mechanism of injury, and age. Importantly, the missed therapeutic opportunity, and care inequity, persisted throughout ED admission with many patients in these subgroups failing to receive TXA at any time point.

Major haemorrhage guidelines for PH care, NICE guidelines for hospital practice and TARN criteria for recording eligibility for TXA are all biased towards the more extreme clinical signs of bleeding, for example cardiovascular shock, and/or receipt of a blood transfusion being the key indicators for administration. There is, however, evidence that a more liberal use of TXA for any trauma patient at risk of bleeding confers a survival advantage^29^. This study highlights a clear gap between the real-world use of TXA and existing TXA guidelines for managing trauma haemorrhage of any severity. The Bleeding Audit Triage Trauma^30,31^ is a validated score for predicting death from bleeding, and a published Bayesian model for predicting TIC^32,33^, blood transfusion and mortality, are two examples of simplified decision support tools. Both are suitable for use by PH care practitioners, which if rolled out widely, in conjunction with focused education on patient groups at risk of under-treatment, have the potential to improve compliance of early TXA administration to injured patients at risk of bleeding. Specifically, the Bleeding Audit Triage Tool has an age component which if applied at scene may increase treatment in older patients. Generally, paramedics and trauma teams in the ED need greater awareness and training around the patient groups we have identified in this study that are far less likely to receive TXA. There is an unexplained clinical bias to selectively administer TXA to younger patients, injured males, and/or those with more violent or higher energy mechanisms of injury and should be urgently addressed to ensure equity of care. Further work should examine decision-making and clinician behaviours around administration of TXA, or the unconscious decision to withhold life-saving treatment for bleeding in trauma.

The study has a number of limitations. First, not all ambulances or fast response vehicles that arrive on scene have a paramedic, for example technician only crews and therefore are unable to administer TXA. The TARN dataset does not provide this level of information and is a likely confounder in the rate of PH TXA use for patients with low energy mechanisms, for example falls less than 2 m who are more likely to receive a lower triage category to which nonparamedic crews will be dispatched. Any confounding; however, cannot explain the similar trends observed in ED TXA use. Second, we are unable to determine from the retrospective dataset the decision-making process, or rationale of paramedics and physicians at the ED trauma call. In particular, we cannot say why practitioners deviated from national guidelines for the treatment of major haemorrhage with TXA, or why it was selectively applied to particular patient cohorts. Behavioural studies of clinicians are required to further examine and understand the subjective decision-making of PH care providers and how these are influenced by patient and injury factors. Relevant contraindications to TXA use in trauma are thromboembolic disease and a history of convulsions, neither of which are easily discernible during the clinical examination or patient history, and therefore we consider the risk of bias from an active clinical choice to withhold treatment based on these factors to be low. TARN does now collect information on patient comorbidities but these were not available in our dataset and medication history is not reported; therefore, we are unable to determine the impact of these factors on the treatment compliance. Third, similarly from the dataset it was possible to understand the impact of dynamic physiology on treatment decisions as TARN only collects the first set of PH observations. If the first set of vital signs are not complete or not available, then ED observations are permissible, which may not represent the clinical status during which decisions to administer PH TXA were made by the clinician. Fourth, the TARN recording of TXA administration does not describe which PH provider administered the medication, for example first paramedic on scene or a PH doctor, only that a HEMS service was present. Fifth, national guidance for TXA dosing was to follow the CRASH-2 trial protocol (1 g bolus then 1 g infusion) but we were unable to explore different doses that may have been given, intentionally or in error, within the context of this study due to the available data. Sixth, TARN excludes PH deaths and at present there is no national repository of medical records for this patient cohort who die before reaching the hospital. Seventh, we were not able to look at clinical outcomes as TARN does not capture all deaths in patients who may have received TXA, for example PH deaths and other important outcomes, for example venous thromboembolism are not mandatory fields for reporting. Finally, TXA eligibility according to TARN best practice tariff was modified over the duration of the study towards a more liberal approach over time and it was not possible to specifically determine the impact of these changes although NICE and JRCALC guidelines have remained constant.

In summary, since the adoption of TXA into hospital and PH national guidelines there has been a threefold increase in use for injured patients. Time to treatment has reduced although significant numbers of patients continue to receive TXA late, or not at all. Practice guidelines for the use of TXA are not universally applied across the spectrum of older trauma patients, those with low energy mechanism of injury, and the nonshocked cohort significantly less likely to receive it. Our data suggests that PH ambulance crews may not associate this type of patient as one at risk of haemorrhage. Paramedics all carry TXA and are often the first on scene of an incident, therefore need greater awareness of the patient subgroups identified in this study who are less likely to treated PH, given it is one of the few interventions in their kit that is proven to improve survival from bleeding. Physician led teams had improved compliance with TXA treatment guidelines suggesting that targeted education for ambulance crews may be of importance in achieving widespread implementation. An alternative mode of delivery, for example intramuscular has been evaluated in small studies for bleeding patients^34^ and in mild traumatic brain injury is under evaluated in the CRASH-4 clinical trial (NCT04521881). This method of dosing may improve compliance for those bleeding patients in which TXA treatment is not currently possible due to inability to secure venous access by paramedics, or providers at scene that do not have the necessary skill set to administer intravenous medication. Key performance indicators for PH practitioners, training and a greater understanding of early decision-making are needed to address the barriers to universal early TXA for all injured patients with bleeding.

## Ethical approval

Not required. National trauma registry with anonymised data.

## Consent

Not applicable.

## Sources of funding

Not applicable.

## Author contribution

Conceptualization: K.Band R.D.; Data curation: A.A.; Formal analysis: A.A., E.C., A.R., and R.D.; Funding acquisition and investigation: A.A. and R.D.; Methodology: A.A., E.C., and R.D.; Project administration and resources: K.B.; Software and supervision: E.C., K.B., and R.D.; Validation: R.D.; Visualisation: A.A. and A.R.; Writing – original draft: A.A., E.C., and R.D.; Writing – review and editing: A.A., E.C., A.R., K.B., and R.D.

## Conflicts of interest disclosure

RD receives departmental support for consumables and equipment from Werfen (point of care coagulation device manufacturer). The remaining authors declare no conflicts of interest.

## Research registration unique identifying number (UIN)

Research Registry. UIN 8916 https://www.researchregistry.com/browse-theregistry#home/registrationdetails/644aeb1a4be00300294c1325/.

## Guarantor

Ross Davenport.

## Provenance and peer review

Not commissioned, no funding. Externally peer-reviewed.

## Data availability statement

Data sharing: patient level data are available, subject to a standard data sharing agreement (which can be found at www.tarn.ac.uk), from the corresponding author at ross.davenport@qmul.ac.uk. Individual participant consent for data sharing was not obtained, but the presented data are anonymised, risk of identification is low and approval is in place from the UK Department of Health’s Patient Information Advisory Group.

## Supplementary Material

SUPPLEMENTARY MATERIAL
