# Supplementary material for: Nationwide analysis of prehospital tranexamic acid for trauma demonstrates systematic bias in adherence to treatment guidelines: a retrospective cohort study
Source: Int J Surg. 2023 Sep 15;109(12):3796–803. doi: 10.1097/JS9.0000000000000712 (PMC10720772; doi:10.1097/JS9.0000000000000712)
Supplement: SUPPLEMENTARY MATERIAL [file js9-109-3796-s001.docx]

Supplemental Table 1: List of haemorrhage control operation and interventions used to identify submissions

| **Operations:** | **Interventions:** |
| --- | --- |
| Abdominal Packing | Direct Compression |
| Amputation of Upper/Lower Limb | Embolisation |
| Aortic Repair |  |
| BOAST 4 – External fixation – Circular frame |  |
| BOAST 4 – External fixation – Monolateral (Non-circular) frame |  |
| BOAST 4 – Internal fixation: Nail |  |
| BOAST 4 – Internal fixation: Other |  |
| BOAST 4 – Internal fixation: Plate |  |
| BOAST 4 – POP |  |
| Bowel operations (specified) |  |
| Caesarian Delivery |  |
| Cholecystectomy |  |
| Diaphragm repair |  |
| Embolisation (interventional radiology) |  |
| Excision of Pancreas |  |
| External Fixation Pelvis |  |
| Extracorporeal Circulation (incl. ECMO) |  |
| Fixation of Acetabulum |  |
| Fixation of Pelvic Ring |  |
| Heart Bypass |  |
| Heart Surgery |  |
| Hemicolectomy/Colectomy |  |
| Ileectomy |  |
| Laparoscopy |  |
| Laparotomy |  |
| Limb perfusion |  |
| Nephrectomy |  |
| Packing of Nose |  |
| Pericardiocentesis |  |
| Pneumonectomy |  |
| REBOA, abdominal |  |
| REBOA, thoracic |  |
| Rectal operation |  |
| Repair Colon laceration |  |
| Repair Kidney laceration |  |
| Repair Liver laceration |  |
| Repair mesentery of colon |  |
| Repair mesentery of small bowel |  |
| Repair Oesophagus |  |
| Repair of Artery |  |
| Repair of chest wall |  |
| Repair of Duodenum |  |
| Repair of Ileum |  |
| Repair of Jejunum |  |
| Repair of lung |  |
| Repair of Stomach |  |
| Repair of Vein |  |
| Repair Rupture to Bladder |  |
| Repair Spleen |  |
| Resection Liver |  |
| Rib fracture fixation |  |
| Splenectomy |  |
| Sternum fixation |  |
| Thoracotomy |  |
| Tracheostomy |  |

Supplemental Table 2: Logistic regression analysis of age and sex for patients receiving PH TXA comprising the interaction between the two independent variables.

|  | **β** | **(95% CI)** | **Odds Ratio** | **(95% CI)** | **p** |
| --- | --- | --- | --- | --- | --- |
| Intercept | 0.410 | (0.206; 0.614) | 1.507 | (1.229; 1.848) | <0.001 |
| Age | -0.032 | (-0.036; -0.028) | 0.969 | (0.965; 0.972) | <0.001 |
| sex, male | -0.393 | (-0.620; -0.166) | 0.675 | (0.538; 0.847) | <0.001 |
| Age*sex | 0.013 | (0.009; 0.017) | 1.013 | (1.009; 1.017) | <0.001 |
