# Supplementary material for: Nationwide analysis of prehospital tranexamic acid for trauma demonstrates systematic bias in adherence to treatment guidelines: a retrospective cohort study
Source: Int J Surg. 2023 Sep 15;109(12):3796–803. doi: 10.1097/JS9.0000000000000712 (PMC10720772; doi:10.1097/JS9.0000000000000712)
Supplement: SUPPLEMENTARY MATERIAL [file js9-109-3796-s002.docx]

Supplemental figure:

*Figure 1A: Cumulative TXA use in 15-minute windows for TXA eligible patients from 2017 to 2019; 1B: Cumulative TXA use in 15-minute windows for TXA eligible shocked patients from 2017 to 2019; 1C: Time from injury to PH TXA administration stratified by mechanism of injury in shocked & RBC transfusion patients. 1D: Time from injury to ED TXA administration stratified by mechanism of injury in shocked & RBC transfusion patients. *signifies p value <0.001 when comparing time from injury to TXA in penetrating vs other types of mechanism of injury.*
